# Systemic virus distribution and host responses in brain and intestine of chickens infected with low pathogenic or high pathogenic avian influenza virus

**DOI:** 10.1186/1743-422X-9-61

**Published:** 2012-03-06

**Authors:** Jacob Post, Dave W Burt, Jan BWJ Cornelissen, Venice Broks, Diana van Zoelen, Ben Peeters, Johanna MJ Rebel

**Affiliations:** 1 Central Veterinary Institute of Wageningen UR, P.O. Box 65, 8200 AB Lelystad, The Netherlands; 2 The Roslin Institute and Royal (Dick) School of Veterinary Studies, Division of Genetics & Genomics, University of Edinburgh, Edinburgh, UK

**Keywords:** Avian Influenza, Chickens, Gene expression, Systemic distribution

## Abstract

**Background:**

Avian influenza virus (AIV) is classified into two pathotypes, low pathogenic (LP) and high pathogenic (HP), based on virulence in chickens.

Differences in pathogenicity between HPAIV and LPAIV might eventually be related to specific characteristics of strains, tissue tropism and host responses.

**Methods:**

To study differences in disease development between HPAIV and LPAIV, we examined the first appearance and eventual load of viral RNA in multiple organs as well as host responses in brain and intestine of chickens infected with two closely related H7N1 HPAIV or LPAIV strains.

**Results:**

Both H7N1 HPAIV and LPAIV spread systemically in chickens after a combined intranasal/intratracheal inoculation. In brain, large differences in viral RNA load and host gene expression were found between H7N1 HPAIV and LPAIV infected chickens. Chicken embryo brain cell culture studies revealed that both HPAIV and LPAIV could infect cultivated embryonic brain cells, but in accordance with the absence of the necessary proteases, replication of LPAIV was limited. Furthermore, TUNEL assay indicated apoptosis in brain of HPAIV infected chickens only. In intestine, where endoproteases that cleave HA of LPAIV are available, we found minimal differences in the amount of viral RNA and a large overlap in the transcriptional responses between HPAIV and LPAIV infected chickens. Interestingly, brain and ileum differed clearly in the cellular pathways that were regulated upon an AI infection.

**Conclusions:**

Although both H7N1 HPAIV and LPAIV RNA was detected in a broad range of tissues beyond the respiratory and gastrointestinal tract, our observations indicate that differences in pathogenicity and mortality between HPAIV and LPAIV could originate from differences in virus replication and the resulting host responses in vital organs like the brain.

## Background

Despite enormous efforts in the last decades, type A influenza viruses are still a serious threat to human health. Influenza A viruses have been isolated from various animals including birds, pigs, horses and sea mammals. Isolates are classified by the viral surface antigens hemagglutinin (HA) and neuraminidase (NA).

Work, started in the late seventies, revealed the important role of HA cleavage for the pathogenicity of (avian) influenza viruses [1]. HA cleavage is a prerequisite for fusion of the viral and endosomal membranes and therefore for viral infectivity [2]. The direct link between HA cleavage and viral reproduction makes endoproteases the major determinant for viral distribution, pathogenicity and mortality. Low pathogen avian influenza viruses (LPAIV) possess a single arginine residue at the cleavage site favoring trypsin-like proteases, which are thought to be secreted only by cells of the respiratory and intestinal tract. The most common sequence of the HA cleavage site of viruses with high pathogenicity (HPAIV) consist of a polybasic cleavage site, which can be recognized and cleaved by both trypsin- and ubiquitously distributed subtilisin-like proteases [3]. For chicken, the above formulated model of HPAIV and LPAIV distribution, was supported by many studies [4], but is not without any debate. First, high pathogenicity is not exclusively linked to the HPAIV cleavage motif [5-7] or degree of HA cleavability [8]. Second, the model is at least incomplete for ducks [9] and finally, some LPAIV strains were found systemically in organs beyond the respiratory and intestinal tract [10-15].

Albeit LPAIV strains can replicate intensively in chicken organs of the respiratory and intestinal tract, the mortality is low. Whether differences in disease development and mortality between HPAIV and LPAIV are due to differences in tissue tropism or host responses is currently unknown.

To investigate in more detail the differences in disease development of high- and low pathogenic influenza virus field isolates in chickens, two genetically closely related HPAIV and LPAIV variants of H7N1 [16,17] were used in combination with a quantitative RT-PCR. Special attention was given to intestine and brain because both these organs are infected, but differ in the availability of the LPAIV specific cleavage proteases. Although trypsin-like proteases are found in the human brain [18], trypsin-like proteases are thought to be absent in the chicken brain [19]. To measure differences in host responses a genome-wide Affymetrix array was used to measure gene expression at different time points during infection.

## Methods

### Viruses

H7N1 Avian influenza virus strains HPAIV (A/turkey/Italy/4580/99) and LPAIV (A/chicken/Italy/1067/99) with an intravenous pathogenicity index (IVPI) of 3.0 and 0.0 respectively, were obtained from Dr. Ilaria Capua (Istituto Zooprofilattico Sperimentale delle Venezie, Italy). The strains are genetically closely related, with the major differences in genes that are related to replication opportunities of the virus [[16,17], B. Peeters, pers. comm.]. Accession nr. are: CY095506-CY095512 for H7N1 LPAIV and CY021405- CY021412 for H7N1 HPAIV. The viruses were propagated and titrated in the allantoic cavities of 10-day-old specific pathogen free embryonated chicken eggs to prepare stock virus. For animal experiments, virus was diluted in sterile phosphate buffered saline (PBS) to 10^6 ^EID_50_/ml immediately prior to use. For infection of chicken embryo brain cell cultures, the stocks were diluted in culture medium.

### Animal experiments

#### Chickens

Lohmann brown male layers were obtained from a commercial breeder (Pronk's Broederij, Meppel, The Netherlands). After hatch, the chickens were housed in floor cages for 3 weeks without any immunization. For each experiment, chickens were randomly distributed over two treatment groups. Feed and water were provided *ad libitum*. All studies were approved by the institutional Animal Experiment Commission in accordance with the Dutch regulations on animal experimentation.

#### Experiment I

Chickens of group 1 were inoculated with 0.2 ml (2*10^5 ^EID_50_) of the H7N1 HPAIV strain, equally divided between the intranasal and intratracheal route. In the same way, animals of group 2 received 2*10^5 ^EID_50 _of the H7N1 LPAIV strain. A control group of chickens was inoculated with 0.2 ml PBS.

#### Experiment II

Chickens of group A were treated exactly as group 1 (HPAIV) from exp. I. The chickens of group B were inoculated with 0.2 ml PBS (controls).

#### Experiment III

Chickens of group C were treated exactly as group 2 (LPAIV) from exp. I. The chickens of group D were inoculated with 0.2 ml PBS (controls).

#### Sampling

Six chickens from each group were sacrificed just before infection (t = 0) and at 4, 8, 16 and 24 h post infection (h.p.i.) (Exp I) at 1, 2 and 3 days post infection (d.p.i.) (Exp. II) or at 1, 2, 4 and 7 d.p.i. (Exp. III). From all sacrificed chickens, gross pathology of the organs was studied. Blood (PBMC), lung (caudal), trachea (medial), ileum (adjacent to the meckel's diverticulum) and forebrain were collected. For Exp. III also spleen (medial) was collected. For RNA isolation, organs were snap-frozen in liquid nitrogen and stored at -80°C until use. All HPAIV experiments were performed in Biosafety Level 3 facilities.

### Detection of viral RNA

#### RNA isolation

Frozen samples were homogenized (Pro2000 homogeniser) in Trizol (Invitrogen) according to the manufacturer's instructions with minor modifications. Subsequently, a phase separation with chloroform was performed and RNA was precipitated using 2-propanol. For microarray analysis, additional purification steps were performed with the Macherey-Nagel NucleoSpin^® ^RNA II kit (April 2007/Rev. 07). With the Agilent Bioanalyzer (lab on chip, Agilent) the quality and integrity of the RNA samples was analyzed.

#### Quantitative PCR assay

RNA from homogenized organs was tested using a one-step quantitative RT-PCR for detection and quantification of the AI matrix gene [20]. Data were expressed as Ct value and compared to a known standard curve. Standard precautions designed to prevent contamination during qPCR were followed. A control group of samples from uninfected animals was included in each run. Because no Ct-values were found for any organ of control chickens at all-time points tested during a 45 cycles run with this PCR, we considered all Ct-values ≤ 45 as positive. Samples negative for viral RNA detection are depicted in figures as Ct = 0.

#### RT-PCR

The relative quantitation of gene expression was carried out using an MX3005 (Stratagene). cDNA was made using random hexamer primers and reverse transcriptase RT-PCR. Primers and probes (Table 1) were designed by TIB MolBiol. Probes were labeled with the reporter dye carboxyfluorescein (FAM) and the quencher tetramethyl-6-carboxyrhodamine (TAMRA). PCR cycling was performed as follows: 95°C for 15 min, followed by 45 cycles of 95°C for 5 s, 50°C for 20 s and 72°C for 20 s. Threshold values were set at a standard value (0.1), which corresponded generally to the midway point of the amplification plots. Relative expression values were normalized against 28S rRNA [21]. For the quantification, a standard curve of the plasmid with the insert of the gene of interest, constructed in pGEM-T easy (Promega) was used.

**Table 1:** RT-PCR primer and probe sequences

Target gene	Primer/ Probe	Sequence	Accession **no**.
FKBP5	Forward primer	5'- CAAAGAGTCATGGGAGATGG - 3'	NM_001005431
	Reverse primer	5'- ATCAGAGGCTTTCGACTCCT - 3'	
	Probe	5'- 6FAM-TCCTGTCCCGCTCGTTGTGC-TMR	
PER2	Forward primer	5'- TCCCAACTATACGGAGGACA - 3'	NM_204262
	Reverse primer	5'- AAGTGTTCGTGTGAGCCATT - 3'	
	Probe	5'- 6FAM-CACCCCAGTGTTCAGGAGATCACA-TMR	
STC2	Forward primer	5'- AGGTCTAGCTGCGTTCTGTG - 3'	XM_414534
	Reverse primer	5'- TGGTTCGAGCTTGTTCTACC - 3'	
	Probe	5'- 6FAM-AAGGCTGCCCTGACCCAAGG-TMR	

### Virus isolation and detection

The presence of H7N1 LPAIV in brain and lung samples (experiment III, 4 d.p.i.), was tested by the method of Hirst [22] and confirmed by qPCR. Lung was taken for its known sensitivity for LPAIV.

#### In ovo injection

Approximately 200 mg tissue sample was homogenized in 2 ml PBS and clarified (30 min 1900 x*g*). This method has been previously tested and appeared to have no effect on the titer of the virus (data not shown). Five 10-days-old embryonated eggs were inoculated each with 200 μl of the supernatant and incubated for 7 days or until embryonic death. The allantoic fluid was harvested, clarified and the supernatant was stored at -80°C until use.

#### HA-assay

Briefly, two-fold serial dilutions of allantois fluid in PBS were incubated with a 1% suspension of chicken erythrocytes for 45 min at 4°C. The hemagglutination titer is defined as the reciprocal value of the highest (log_2_) dilution of allantois fluid that causes visible erythrocyte agglutination.

### Brain cell culture

The method of preparing chick embryo brain cell cultures (CEBCC) was adapted from the work of Sato et al. [23] and Parker et al. [24].

#### Primary culture

Brains were isolated from 14-day-old chicken embryo's. The brains were washed with sterile PBS and trypsinised (0.25%) for three times 3 min at room temperature with a magnetic stirrer. After each trypsin incubation, cells were isolated and suspended in FCS. The suspension was filtered through a 100 μm cell strainer, centrifuged (10 min 450 x*g*) and resuspended in 150 ml GMEM/EMEM (1:1) culture medium supplemented with 10% FCS and 2% antibiotic mix. The cell suspension was divided over 150 cm^2 ^flasks. Cells were incubated for 48 h in an humidified incubator at 37°C.

#### Secondary culture

The confluent layer of primary cells was washed with sterile PBS and incubated with 0.25% trypsin for 5 min at 37°C. The trypsin was inactivated with FCS, cells were harvested and the suspension was pelleted (10 min 450 x*g*). The cells originating from one flask were suspended in 50 ml culture medium supplemented with 5% FCS and 2% antibiotics. Finally, the suspension was divided over 24 wells culture plates. After incubation the supernatant was removed and plates were stored at -80°C until screening.

The presence of astrocytes in the CEBCC was detected with an slightly adapted immunoperoxidase monolayer assay (IPMA) according to Wellenberg et al. [25]. Briefly, the cell monolayer's were fixed with 4% formaldehyde. Endogenous peroxidase activity was blocked with 1% hydrogen peroxide in methanol. After extensive washing with PBS, the cells were incubated with anti GFAP, to detect astrocytes (Millipore). The cells were washed and incubated with RaMPO (Dako). Binding of the antibody was visualized with AEC. Finally, the plates were washed with tap water and dried. Plates were examined using a Leica DFC 420 microscope (Leica).

#### Virus detection

Virus was detected with an antibody against the nucleoprotein of influenza A (HB65, ATCC) using the IPMA protocol as described above. In case of the immunofluorescent double staining, HB65 (IgG_2a_) and anti-GFAP (IgG_1_) were visualized with Alexa Fluor 594 (a. IgG_2a_) and Alexa Fluor 350 (a. IgG_1_).

#### Experiment A

Secondary cultures were incubated in duplicate with culture medium, 10^6 ^EID_50 _H7N1 LPAIV in culture medium or 10^6 ^EID_50 _H7N1 HPAIV in culture medium for 24 h at 37°C.

#### Experiment B

Secondary cultures were incubated for 2 h with 10^6 ^EID_50 _H7N1 LPAIV or HPAIV. Supernatant was collected, the cells were washed with PBS and one plate was stored at -80°C (T = 0 h). To a complementary plate, fresh culture medium was added. After incubation in an humidified incubator at 37°C for 24 h, the supernatant was collected (S24h). The cells were washed and the plate was stored at -80°C (T = 24 h). Fresh secondary cultures were incubated for 24 h with 10% S24h supernatant, with or without 0.005% trypsin. Presence of the virus in the S24h supernatant was confirmed by qPCR.

### Gene regulation

Early changes in AI-induced gene expression levels were analyzed using brain and ileum samples of Exp. I at 0, 4, 8 and 16 h.p.i. Samples from experiment I were taken since in this experiment the LPAIV and HPAIV infection could be compared in the same experimental setting, meaning that less variation in chickens due to experimental design, hatching and housing conditions or genetic background was expected. Five individual birds were used for each time point.

#### RNA labeling

The Affymetrix One Cycle Target Labeling Kit was used to synthesize the biotin-cRNA. Labeling of 20 μg of RNA, hybridization, staining, washing steps, and array scanning were carried out at the Roslin institute ARK (Edinburgh, UK) according to standard protocols http://www.ark-genomics.org/protocols.

#### Microarray hybridization

Biotinylated fragmented cRNA was hybridized to the Affymetrix Chicken Genome Array. This array contains comprehensive coverage of 32,773 transcripts corresponding to over 28,000 chicken genes. The Chicken Genome Array also contains 689 probe sets for detecting 684 transcripts from 17 avian viruses. Experimental groups were control brain and ileum tissues from (non-infected), low LPAI or HPAI infected birds samples at 0, 4, 8 or 16 h.p.i. For each experimental group, 4 out of 6 biological replicates were hybridized. Hybridization was performed at 45°C for 16 h in a hybridization oven with constant rotation (60 rpm). The microarrays were then automatically washed and stained with streptavidin-phycoerythrin conjugate (SAPE; Invitrogen, Paisley, UK) in a Genechip Fluidics Station (Affymetrix). Fluorescence intensities were scanned with a GeneArray Scanner 3000 (Affymetrix). The scanned images were inspected and analyzed using established quality control measures. Array data have been submitted to Array Express under accession number E-MEXP-3109.

#### Statistical analysis and detection of differentially expressed genes

Gene expression data generated from the GeneChip Operating Software (GCOS) was normalized using the PLIER (probe logarithmic intensity error) method [26] within the Affymetrix Expression Console software package. This normaliz.ed data was then log2 transformed (log2(exprs(eset) + 16)) and then analyzed using the limma and FARMS [27] packages within R in Bioconductor [28]. Probes with a FDR value < 0.05 were considered significantly different. From these, genes with a fold change ≥ 2 were analyzed. Comparisons were made between experimental groups to test for host responses (LPAI vs. control, HPAI vs. control).

#### Analysis of differentially expressed genes

Gene symbols were assigned to chicken genes using orthologous relationships from the Ensembl Compara database using the BioMart tool http://www.ensembl.org/biomart. In this way, information from the human orthologues was transferred to the chicken genes. In order to determine which biological pathways are involved in these responses to viral infection, the differentially expressed gene sets were analyzed using Pathway Express http://vortex.cs.wayne.edu Software compared to genes that did not respond to treatments. Over representation of specific pathways was tested using the hypergeometric distribution and FDR was used to correct for multiple testing of all pathways. Expander software http://acgt.cs.tau.ac.il/expander enabled the data to be clustered using CLICK method and analyzed for enriched GO-terms and transcription factor binding sites. Normalized and log2 transformed data was filtered further to extract genes showing variation across experimental treatments (> 0.5 standard deviation). Further analysis of Gene Ontology terms was made using Ontologizer http://compbio.charite.de/index.php/ontologizer2.html using Parent-child method and *p*-values were corrected for multiple testing by the Westfall-Young-Single-Step method. Samples from Exp I were used for analysis.

### Apoptosis

Four μm thick brain sections from Exp II & III (2 d.p.i.) were mounted on poly-L-lysine coated slides, subsequently de-waxed by immersion in fresh xylene for 5 min at room temperature, then rehydrated in a graded alcohol series. The *in situ *detection of fragmented DNA using the DeadEnd™ Colorimetric Apoptosis Detection System was performed according to the manufacturer's instructions (Promega).

### Statistical analysis

Comparison of qPCR data on AI load in chicken samples were analyzed for statistical significance by the Mann-Whitney *U *test, with *P *< 0.05 considered significant.

## Results

### Viral distribution

In order to determine the distribution of H7N1 HPAIV and LPAIV over organs of chickens, the presence of viral RNA was tested with the qPCR.

#### HPAIV

**Figure 1:**
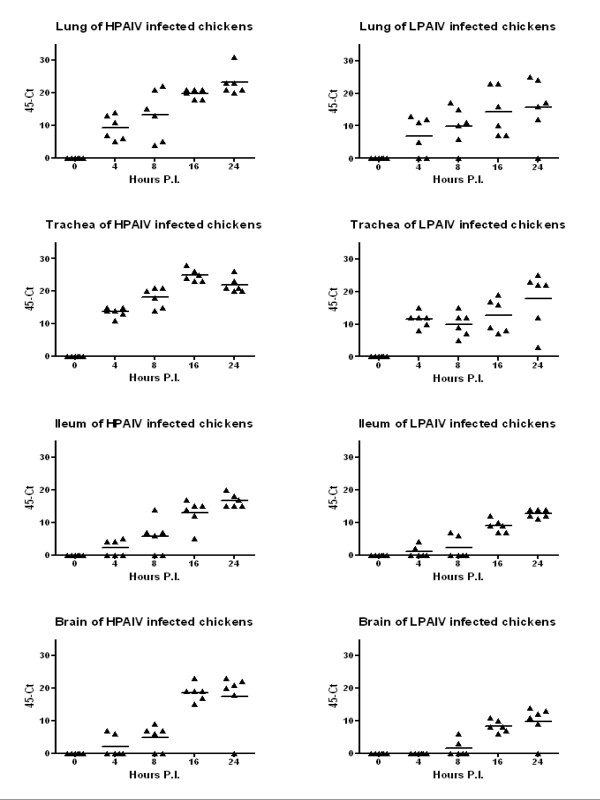
**Scatter plot of 45-Ct values of viral RNA from individual birds in organs at different hours post inoculation (h.p.i.)**. Chickens were inoculated with H7N1 HPAIV or LPAIV and the presence of viral RNA in organs was examined from 0 to 24 h.p.i. (Exp I). The horizontal line represents the mean of 6 individual birds. Triangles represent the Ct-value of individual birds. No Ct-Values were found in controls.

**Figure 2:**
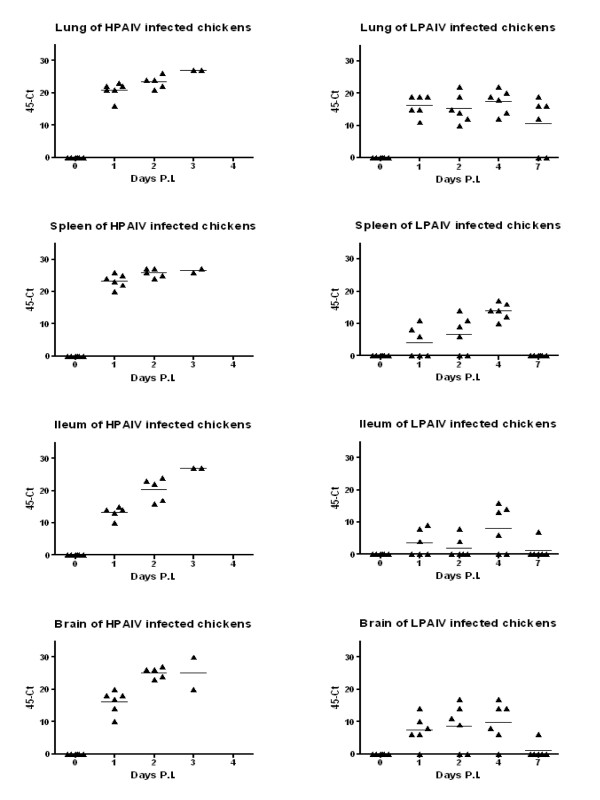
**Scatter plot of 45-Ct values of viral RNA from individual birds in organs at different days post inoculation (D.P.I.)**. Chickens were inoculated with H7N1 HPAIV (Exp II) or H7N1 LPAIV (Exp III) and the presence of viral RNA in organs was examined at different time points. The horizontal line represents the mean of individual birds. Triangles represent the Ct-value of individual birds. When less than 6 triangles are depicted mortality of chickens due to infection had occurred. No Ct-Values were found in controls.

H7N1 HPAIV infected chickens developed clear manifestations of illness with depression and ruffled feathers, leading to death. At day 2 post infection already one of the chickens had died and 3 d.p.i. four out of six had died. qPCR data showed that high amounts of viral RNA were present in all organs tested (Figures 1 & 2). Despite this, differences were seen between organs. A rapid and strong increase of viral RNA was seen within 24 h, especially in the inoculated lung and trachea (Figure 1).

#### LPAIV

No clinical signs were observed during the experimental trial. However, viral RNA was found within 4 h.p.i. in organs of the respiratory and gastrointestinal tract (Figure 1). Eventually, viral RNA was found for a prolonged time in lung, spleen, ileum and brain with the highest load around 4 d.p.i. (Figure 2). While LPAIV RNA could be detected for at least a week in the lung, viral RNA could only incidentally be detected in spleen, ileum and brain at 7 d.p.i. Significant differences between HPAIV and LPAIV RNA load were found for lung (1 and 2 d.p.i.; 0.01 <*p *< 0.05), trachea (8 and 16 h.p.i.; *p *< 0.01), spleen (1 and 2 d.p.i.; *p *< 0.01), ileum (1 and 2 d.p.i.; *p *< 0.01) and brain (16 h.p.i., 1 and 2 d.p.i.; *p *< 0.01).

To test whether infectious LPAIV could be isolated from systemic organs, supernatants from homogenates of chickens inoculated with LPAIV (4 d.p.i., Exp III), were injected in 10-day-old eggs. Virus could be detected by means of an HA-test and qPCR (data not shown) in the allantoic fluid of eggs, injected with supernatant of either lung or brain homogenate. HA titers were found in those chickens with the highest viral RNA load, according to the qPCR data of exp III. Sequencing of the cleavage site confirmed that the harvested virus from the allantoic fluid of eggs, was identical to the inoculated LPAIV. We were not able to detect virus in the homogenate of control chickens.

### Brain cell cultures

From the results of the PCR and virus isolation we concluded that both H7N1 HPAIV and LPAIV could spread beyond the respiratory and gastrointestinal tract. However, whether both viruses can actually replicate in brain tissue is not known. Therefore we used an *in vitro *chicken embryo brain cell culture (CEBCC) system.

**Figure 3:**
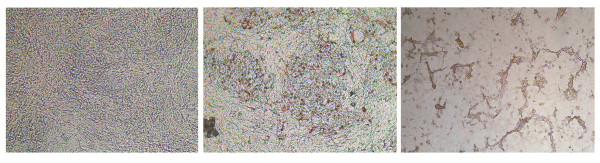
**AIV infected secondary chicken embryonic brain cell cultures stained with the anti-AI nucleoprotein specific Moab HB65**. Cells were cultured for 24 h with Medium (**a**), LPAIV (**b**) or HPAIV (**c**). Magnification: 100×.

**Figure 4:**
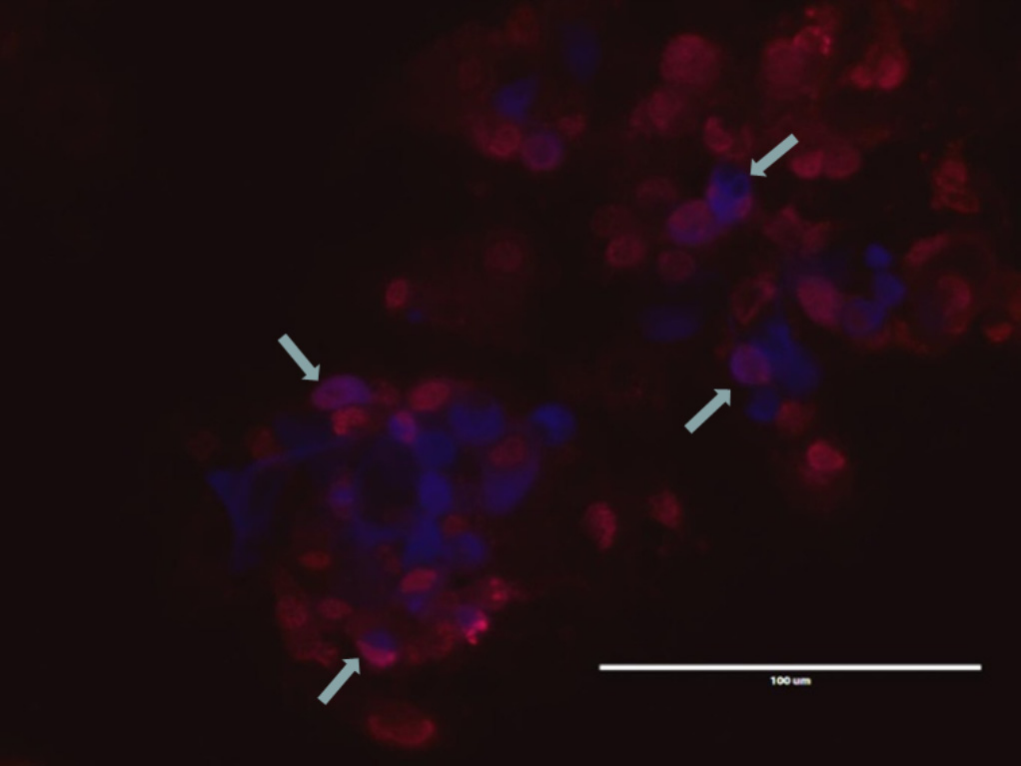
**AIV infected secondary chicken embryonic brain cell cultures**. Staining with anti-GFAP (astrocytes; blue) and HB65 (AI; red). Arrows indicate double stained cells. Bar = 100 μm.

Incubation of the secondary cultures with H7N1 LPAIV, revealed that typical cell clusters were infected by the virus (Figure 3b). With H7N1 HPAIV, cytopathogenic effects (CPE) were seen (Figure 3c). With a monoclonal antibody directed against astrocytes (GFAP) a similar staining of the cell clusters was seen, comparable to that of AIV positive cells, stained with HB65 (data not shown). Double staining of GFAP with HB65 revealed that astrocytes could be infected by AIV (Figure 4, double staining). However, not only astrocytes were infected by AIV (Figure 4, single red staining) and not all astrocytes were AIV infected (Figure 4, single blue staining).

**Figure 5:**
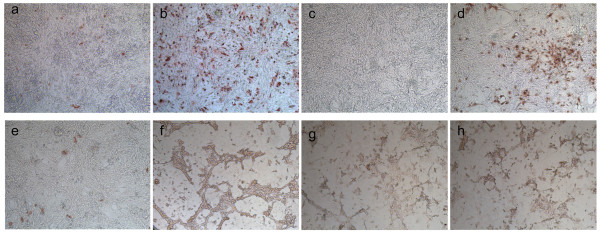
**Secondary chicken embryonic brain cell cultures stained with the anti-AIV nucleoprotein specific Moab HB65**. Cells were cultured with LPAIV (**a**-**d**) or HPAIV (**e**-**h**) for 2 h, washed and incubated with fresh medium for 0 h. (**a**,**e**) or 24 h (**b**,**f**). From the 24 h culture 10% supernatant was transferred to fresh cultures and cultures were incubated for another 24 h without (**c**,**g**) or with (**d**,**h**) 0.005% trypsin. Magnification: 100×.

After 2 h of incubation with 10^6 ^EID_50 _HPAIV or LPAIV, some cells of the CEBC culture were already found positive for AI (Figure 5a/e). After removal of the virus, extensive replication of LPAIV in the cultures was observed at 24 h.p.i. (Figure 5b). Although large amounts of viral RNA (as measured with the qPCR; data not shown) was present in the supernatant of these 24 h cultures, the virus was not able to infect fresh CEBCC (Figure 5c). After adding 0.005% trypsin, the virus present in this supernatant could however infect fresh CEBCC (Figure 5d). In contrast to H7N1 LPAIV, cytopathogenic effects (CPE) were found in all cultures infected with HPAIV or with supernatant from HPAIV infected CEBCC (Figure 5f-h), indicating that HPAIV did not require the addition of trypsin to become infectious.

### Host response

To investigate if chickens react differently to a H7N1 HPAIV or LPAIV infection in infected tissues we studied the ileum and brain of HPAIV and LPAIV infected birds. To investigate organisms with poorly annotated genomes, like poultry, this approach is a challenge. The background is often the species of interest, but when annotation is lacking or poor for a species, identifiers as well as the background can be mapped to human or mice. These species have better annotation of genes and therefore the outcome of the analyses will contain more biological information. Therefore we used For functional analyses human orthologues in a human background.

A disadvantage is that chicken specific genes and processes could not be studied with this approach. Cumulative data of 4, 8 and 16 h.p.i. showed that in brains of LPAIV infected chickens, 4 genes (PER2 http://www.genecards.org/cgi-bin/carddisp.pl?gene=PER2&search, UBC http://www.genecards.org/cgi-bin/carddisp.pl?gene=UBC&search, STC2 http://www.genecards.org/cgi-bin/carddisp.pl?gene=STC2&search, and FKBP5 http://www.genecards.org/cgi-bin/carddisp.pl?gene=FKBP5&search) were significantly down regulated with an at least two fold change, compared to non-infected animals (Table 2). PER2 was already down regulated at 8 h.p.i.

**Table 2:** Microarray data

		T = 4 h	T = 8 h	T = 16 h
Brain	LPAIV	0	1	4
	HPAIV	2	1	4100
Ileum	LPAIV	612	769	739
	HPAIV	687	1434	1307

Numbers represent amount of genes with significant changes in expression level compared to control chickens (T = 0 h)

In HPAIV infected brain samples, approximately 4100 genes were significantly differentially regulated at 16 h.p.i., including the 4 genes found after an LPAIV infection (Table 2). Genes that were affected by HPAIV were mainly part of the Phosphatidylinositol signaling system or the Toll-like receptor signaling pathway (Table 3). In general, the Phosphatidylinositol signaling system was up regulated, while the Toll-like receptor signaling pathway was down regulated.

**Table 3:** Microarray pathway analysis data of brain of chicken infected with HPAIV (16 h.p.i.)

Pathway Name	Impact Factor	Corrected gamma *p*-value
Phosphatidylinositol signaling system	10.701	0.010
Toll-like receptor signaling pathway	9.685	0.013

Numbers represent impact factor of genes within pathways compared to control chickens (T = 0 h)

Compared to the genes that were regulated by HPAIV in the brain, the response in the ileum develops quite differently. Responses develop much faster, but fewer genes were eventually regulated in the ileum at 16 h.p.i. (Table 2). Second, compared to brain different pathways were affected in the ileum after an AI infection (Table 4).

**Table 4:** Microarray pathway analysis data of ileum of chicken infected with LPAIV or HPAIV (8 h.p.i.)

Pathway Name	Impact Factor	Corrected gamma *p*-value
**LPAIV**		
Cell adhesion molecules (CAMs)	368.98	1.60E-156
Phosphatidylinositol signaling system	48.03	2.58E-18
Cell cycle	22.173	1.38E-07
Circadian rhythm	18.024	5.37E-06
DNA replication	13.484	3.07E-04
Homologous recombination	8.473	0.025
**HPAIV**		
Cell adhesion molecules (CAMs)	96.859	7.32E-39
Phosphatidylinositol signaling system	21.192	4.03E-07
Cell cycle	9.921	0.009
Circadian rhythm	75.34	6.33E-30
DNA replication	9.477	0.012
Adherents junction	15.163	9.14E-05

Numbers represent impact factor of genes within pathways compared to control chickens (T = 0 h)

The difference in gene regulation between LPAIV and HPAIV infected chickens was less pronounced in ileum, since responses overlapped largely. Although more genes were found to be induced in ileum after an HPAIV infection compared to an LPAIV infection, pathway analyses revealed that similar pathways are induced in the ileum of LPAIV and HPAIV infected chickens. No effect was seen on PER2, UBC, STC2, and FKBP5. In general, the direction of regulation was less obvious. The majority of genes of the Cell cycle pathway were up regulated, while a number of genes of the Circadian rhythm system were down regulation. Genes of other systems were up- or down regulated.

#### PCR

**Figure 6:**
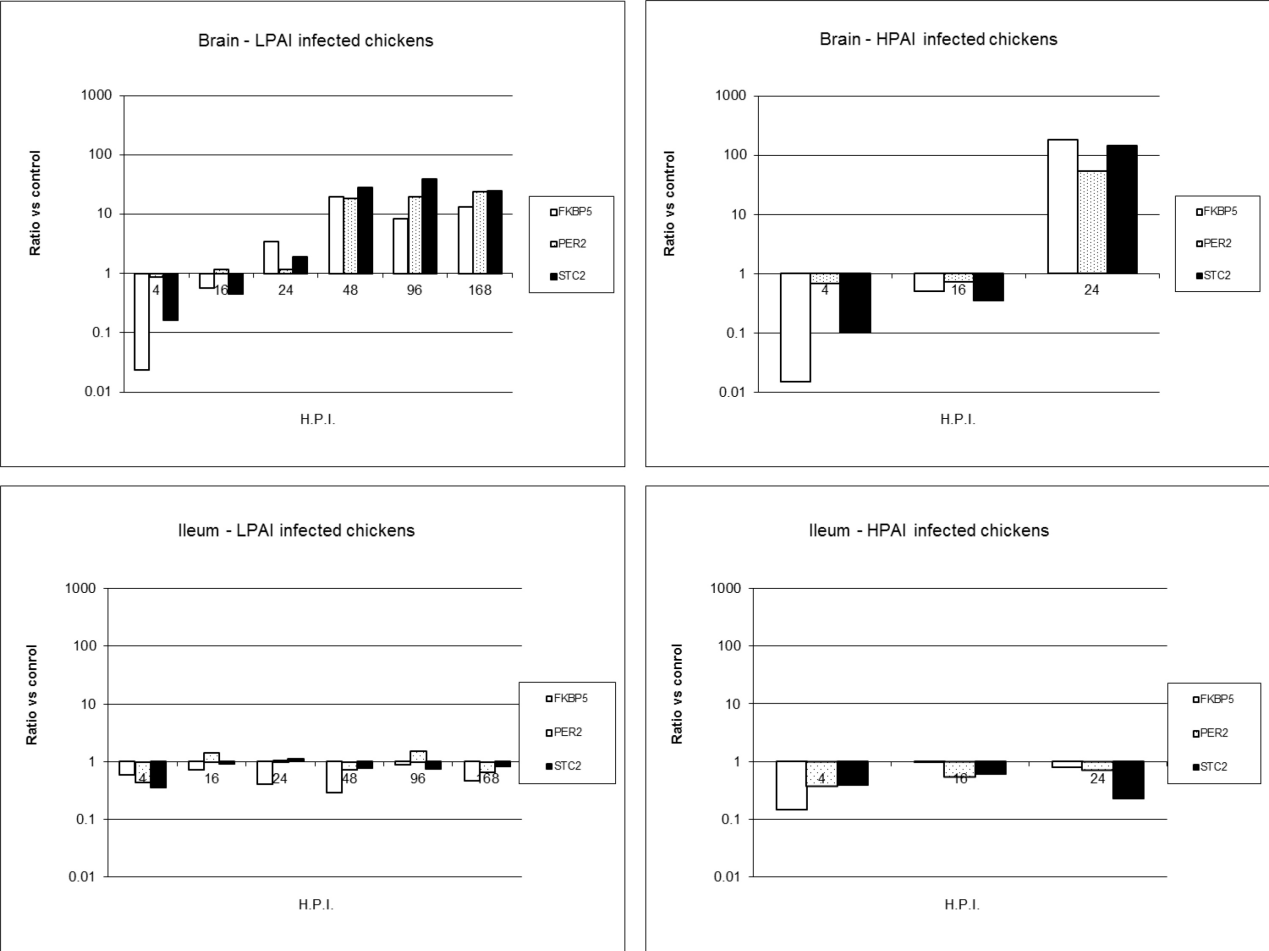
**FKBP5, PER2 and STC2 in AI infected chickens**. Changes in gene expression levels in brain and ileum of chickens infected with AI (Exp I & III). Expression levels of genes were normalized against 28S and compared to normalized data of control chickens as a ratio vs. control.

Three genes that were altered after an LPAIV and HPAIV infection in brain were used to validate microarray responses of LPAIV and HPAIV in brain and ileum (PER2, STC2 and FKBP5) in the experiments I & III. In brain a down regulation of the measured genes, directly after infection, was generally followed by an up regulation (Figure 6). The down regulation early in the infection did confirm the microarray data. The maximal increase was between 10 to 100 fold for LPAIV infected chickens and around 100 fold for HPAIV infected chickens. In accordance with the microarray data, only minor effects were noted on the regulation of these genes in the ileum of H7N1 infected chickens.

### Apoptosis

**Figure 7:**
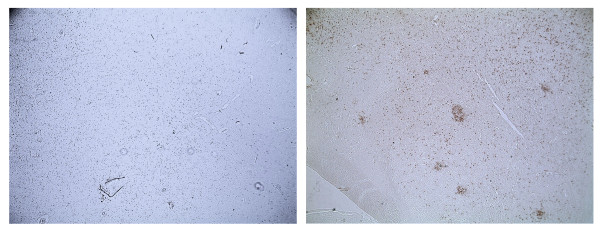
**TUNEL staining of chicken brain 2 d.p.i. a**: H7N1 LPAIV infected chicken; **b**: H7N1 HPAIV infected chicken. Magnification: 100×.

Specific DNA fragmentation was seen in the brain of HPAIV infected chickens (Figure 7b). With this method no specific DNA fragmentation was seen in LPAIV infected chickens (Figure 7a).

## Discussion

Differences in disease outcome between LPAIV and HPAIV infected chickens are obvious. Less obvious is the exact mechanism behind disease development after HPAIV infection. In humans, a cytokine storm (hypercytokinemia) with devastating consequences for the infected organs is thought to contribute to mortality after high pathogenic H5N1 infections [29,30]. Indeed, the high mortality rate of H1N1 during the 1918 influenza pandemic was related to severe pathobiology of the lungs [31].

In accordance to work on H7N3 [32] no major differences in cytokine mRNA levels in the lungs of chickens between H7N1 HPAIV and LPAIV infections could be found during the first 24 h of infection [33]. In fact, only small pathological differences in lung and trachea were found between the two genetically related, but pathogenically distinct variants. Löndt et al. [34] demonstrated for Pekin ducks, that localization of H5N1 HPAIV in heart and brain tissue preceded death and the fact that these are vital organs correlated well with the finding of rapid mortality after HPAIV infections. So, assuming that differences between H7N1 HPAIV and LPAIV infections might be related to the differences in virus localization, we were surprised by the presence of H7N1 LPAIV RNA in organs beyond the respiratory and gastrointestinal tract, where the necessary proteases to cleavage LPAIV are absent. The observation that H7N1 LPAIV RNA could be detected for a prolonged time in multiple organs of all infected chickens indicated that the virus could spread systemically after an intranasal/intratracheal infection. The systemic spread of LPAIV could not be confirmed by immunohistochemistry (IHC) at 2 d.p.i. While all analyzed organs become positive for the virus nucleoprotein after an HPAIV inoculation virus could only be detected in the lung after an LPAIV infection (data not shown). The discrepancies between the qPCR and IHC could be due to differences in measurement, but are probably due to differences in sensitivity. The presence of H7N1 LP virus in brain was confirmed after inoculation of brain homogenate supernatant from LPAIV infected chickens in embryonated eggs and sequencing. Virus was demonstrated in the allantoic fluid by both HA-assay and qPCR. Furthermore, differentially regulated host gene expression in chickens infected with LPAIV was detected in both ileum and brain.

Thus in two separate experiments H7N1 LPAIV RNA could be found systemically. Although unexpected, our data were in line with findings for H7N1 LPAIV in turkeys by Toffan et al. [14]. They found that besides lung, viral RNA was also detected in breast, thigh and blood. In addition, also H9N2 and H5N2 LPAIV were found in chicken organs that were not related to the respiratory and gastrointestinal tract [12,13,15]. Until recently, however, such observations were considered obscurities. The question arises how the presence of LPAIV (RNA) in organs that were expected to be infected by HPAIV only, are in proportion with the differences in mortality between the two strains.

Although brain and ileum are both infected, differences between these organs exist in the availability of the LPAIV cleavage proteases. Chicken brain lacks the required trypsin-like proteases [19]. In line with the absence of these proteases in brain, the differences in viral RNA load between HPAIV and LPAIV infected chickens were considerable. The differences between HPAIV and LPAIV infected chickens were also visualized by the differences of the amount of affected genes in the brain. Similarly, the availability of proteases for both HPAIV and LPAIV cleavage in the ileum might be responsible for the limited difference in viral RNA load and the large overlap in regulated host genes between HPAIV and LPAIV infected chickens. Taking the data of brain and ileum together this could mean that differences in host responses and possible consequences in pathogenicity and mortality are merely determined by the amount of viral RNA or virus instead of other different characteristics between the strains (e.g. PB1 etc.). This hypothesis is supported by the observation that H7N1 HPAIV and LPAIV were closely related and that the differences between the two strains were predominately caused by the differences in the HA cleavage and nearby glycosylation sites [17].

Apart from the differences between high- and low pathogen virus infections, remarkable differences were seen between their effects on the brain and ileum. Remarkable, because differences between the organs were not only seen in the viral RNA load and amount of genes regulated, but also in the host response pathways that were affected. Despite the considerable viral RNA load in the brain of HPAI infected chickens, only two pathways were found to be significantly affected. For ileum at least six affected pathways could be found after analyses of the expression data. The Phosphatidylinositol signaling system, with was activated in both brain and ileum, is in mice important for signal transduction [35]. The detected up regulation of genes of this pathway, might indicate increased cellular activity. The Toll-like receptor signaling pathway, which was regulated in the brain only, has a possible function in clearing the infection. Down regulation of genes of this pathway in the first 24 h, perhaps caused by elements of the influenza virus itself, might hamper the immunological response against the virus, thereby favoring the infection. In ileum, the pathways that are affected after an AIV infection are more diverse, but might be related to tissue regeneration. Possibly, AIV is a lesser threat to ileum because of the fast regeneration of the villus epithelial cells as suggested for Rotavirus by Snodgrass et al. [36].

Despite the differences in the amount of genes regulated, no remarkable discrepancies between LPAIV and HPAIV infected chickens were found for PER2, STC2 and FKBP5 by qPCR. PER2 is expressed in many brain areas and peripheral tissues of mammals and is generally associated with the circadian rhythm [37]. PER2 is also linked to IFN-gamma regulation since PER2-deficient mice had an impaired IFN-gamma production [38]. In the brain of LPAIV infected chickens PER2 was already down regulated at 8 h.p.i. Whether the down regulation of this gene forms the kick-off for other genes is currently unknown. Little is known about STC2, the paralog of the mammalian counterpart of the fish calcium-regulating hormone STC1, except for the fact that over expression might protect cells from apoptosis [39]. STC1 is expressed in multiple organs modulating the immune/inflammatory response [40]. FKBP5 is expressed in a variety of mouse and human tissues and has been shown to interact with various immune response pathways. In brain immunophilins like FKBP5, are modulators of the cortisol-HPA axis response to stress and related chronic brain disorders [41]. In a ferret AIV infection model, it is suggested that the up-regulation of FKBP5 is a physiological response of lung cells to the increase of glucocorticoid, which facilitates the suppressive effect of glucocorticoid on pro-inflammatory cytokine production [42]. Overall, an AIV infection in chicken brain resulted in regulation of genes that are frequently associated with immune regulatory functions, perhaps favoring infection or preventing tissue damage.

Virus is transported through the body via the blood stream [12,43] and in our experiments PBMC were positive for viral RNA after an HPAIV or LPAIV infection (data not shown). Therefore, we cannot exclude the possibility that the viral RNA that we detected by qPCR resulted from the presence of blood in the organs examined. Furthermore, although viral induced gene expression might take place in the brain [44] the data on host gene regulation might also be derived from endothelial cells in the brain instead of from brain cells itself [45,46]. Therefore, embryonic brain cell cultures were used to evaluate whether H7N1 LPAIV could infect cells derived from embryonic brains.

Brain cell cultures revealed that LPAIV, when grown in eggs, could infect embryonic brain cell cultures including astrocytes. LPAIV could also productively replicate in these brain cell cultures, as was seen by an increase in staining of viral nucleoprotein, after removal of the virus. However, progeny virus produced by these cells could only infect fresh brain cell cultures after treatment with trypsin. This indicates that cleavage by trypsin-like proteases was essential for the virus to become infectious. Since H7N1 LPAIV was grown in eggs, we speculate that it contains cleaved HA and is therefore able to infect secondary brain cell cultures and, to some extent, replicate in those cultures. In the absence of trypsin-like proteases however, the newly formed virus remains arrested in its native form, unable to infect fresh cultures. This hypothesis was supported by experiments with a prolonged culturing time: no additional staining, not to mention cytopathogenic effects, were found when the incubation was prolonged after the first 24 h of incubation (data not shown). By adding trypsin, the arrested virus is cleaved and infectivity is restored. In a similar way the isolated virus from homogenated brain samples might be detected. Since the necessary proteases are absent in the brain, the virus may *in vivo *also be present in an arrested form. Trypsin-like proteases in the allantoic of the embryo might be responsible for the (restored) reproducibility of the virus.

The *in vitro *work above may reflect the *in vivo *situation. Cleaved H7N1 LPAIV from primary infected organs like lung and trachea might infect many different organs via the blood. Massive replication in the organs with trypsin-like proteases, makes the virus easy to detect, while arrested virus, in organs with no or hardly any trypsin-like proteases, remain below the detection levels of relative insensitive assays. This hypothesis is supported by the results of a study in turkeys of Toffan et al. [14]. Albeit not discussed, the sensitivity of the test method appeared to be very important in this study: only RT-PCR demonstrated H7N1 LPAIV RNA in each of the tested organs. Since more LPAIV strains were incidentally detected beyond the respiratory and gastrointestinal tract, we consider this feature not to be restricted to H7N1.

To date, all virulent influenza viruses tested have the ability to induce apoptosis *in vitro *[47]. This led to the hypothesis that apoptosis may contribute to the lethality of the host due to viruses that can replicate in a variety of tissues. Indeed, specific apoptosis in the brain of HPAIV infected chickens correlated well with the fact that only HPAIV could replicate in the brain. Apoptosis was already seen 1 d.p.i., when the first chickens showed signs of illness (data not shown). Therefore infection of brain with HPAIV likely contributes to the rapid mortality after infection.

## Conclusions

Overall, with the development of sensitive tests like qPCR, more LPAI viruses may be identified that spread systemically. However, the arrested replication of LPAIV in brain cultures highlights the specific differences between HPAIV and LPAIV, supporting the model for the correlation between HA-cleavability and infectivity. Concerning H7N1, differences in replication and host responses, especially in the brain, may be the main cause of differences in pathogenesis between H7N1 HPAIV and LPAIV strains in infected chickens.

## Abbreviations

LPAIV: Low pathogen avian influenza; HPAIV: High pathogen avian influenza; H.P.I.: Hours post infection; D.P.I.: Days post infection; CEBCC: Chicken embryo brain cell culture.

## Competing interests

The authors declare that they have no competing interests.

## Authors' contributions

JMJR was responsible for the study design and interpretation of the data. BP participated in the study design. DWB was responsible for the microarray work. JBWJC carried out the tunnel assay. VB carried out the RT-PCRs. DvZ participated in the brain cell cultures. JP was responsible for the animal experiment, carried out the qPCRs and brain cell cultures, participated in the interpretation of the data and drafted the manuscript. All authors read and approved the final manuscript.
